# Ferroelastic domain identification in BiFeO_3_ crystals using Raman spectroscopy

**DOI:** 10.1038/s41598-018-36462-5

**Published:** 2019-01-23

**Authors:** Cameliu Himcinschi, Jan Rix, Christian Röder, Martin Rudolph, Ming-Min Yang, David Rafaja, Jens Kortus, Marin Alexe

**Affiliations:** 1 Institute of Theoretical Physics, TU Bergakademie Freiberg, D-09596 Freiberg, Germany; 2 Institute of Materials Science, TU Bergakademie Freiberg, D-09596 Freiberg, Germany; 3 Department of Physics, University of Warwick, Coventry, CV4 7AL United Kingdom

## Abstract

Multiferroic BiFeO_3_ crystals were investigated by means of micro-Raman spectroscopy using the laser wavelengths of 442 nm (resonant conditions) and 633 nm (non-resonant conditions). The azimuthal angle dependence of the intensity of the Raman modes allowed their symmetry assignment. The experimental data are consistent with a simulation based on Raman tensor formalism. Mixed symmetries were taken into account, considering the orientation of the crystal optic axis along a pseudo-cubic <111> direction. The strong anisotropic intensity variation of some of the polar Raman modes was used for line scans and mappings in order to identify ferroelastic domain patterns. The line scans performed with different excitation wavelengths and hence different information depths indicate a tilt of the domain walls with respect to the sample surface. The domain distribution found by Raman spectroscopy is in very good agreement with the finding of electron back scattering diffraction.

## Introduction

**Figure 1:**
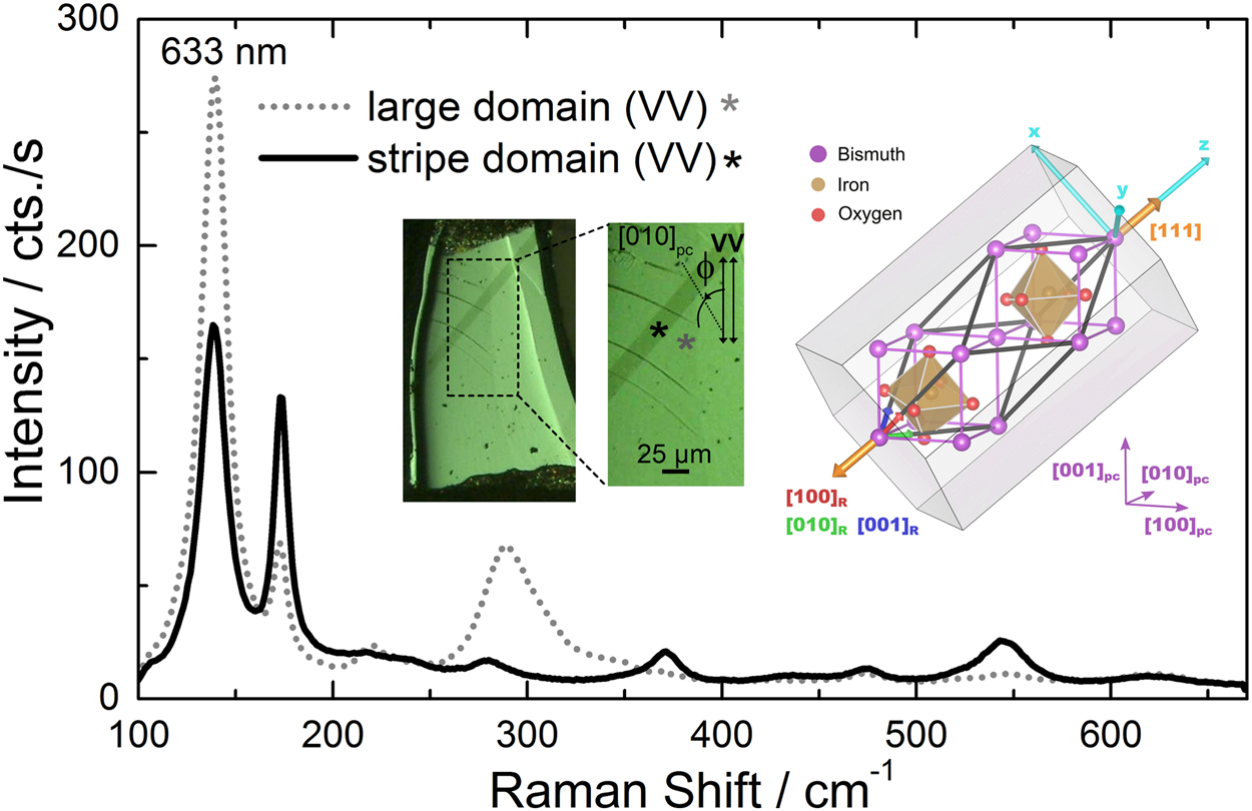
Typical Raman spectra measured in parallel (VV) polarisation using the 633 nm excitation line on the stripe domain (continuous line) and on the large domain (dotted line) of a (001)_pc_ BFO crystal shown in the middle inset of the figure (polarisation microscopy image). Right inset: Alternative structural descriptions of BFO and their crystallographic relationship, using VESTA program^44^. Thick black lines terminate the rhombohedral unit cell (*R3c*). The unit cells plotted in purple and filled by the atoms are pseudo-cubic (*R3m*). The orange arrows mark the polarisation axis. The orthogonal (x,y,z) axes used for the Raman tensors are plotted in cyan. The gray object is an extended hexagonal elementary cell according to Doig *et al*.^45^.

Due to its multiferroic properties at room temperature, the visible-light photovoltaic effect, and related potential applications, BiFeO_3_ (BFO) attracts a lot of scientific interest^1–4^. Under ambient conditions, BFO crystallizes in the trigonal space group (SG) ***R3c*** with the rhombohedral lattice parameters a = 5.6343 Å and α = 59.348°, or alternatively hexagonal lattice parameters a = 5.5876 Å and c = 13.867 Å^5,6^. Neglecting the mutual rotation of neighbouring FeO_6_ octahedrons, the crystal structure of BFO can be approximated by a sheared perovskite structure (see the inset on the right-hand side of Fig. 1) that was reported by Tomashpolskii *et al*.^7^ to crystallize in the space group ***R3m*** and with the lattice parameters a = 3.962 Å and α = 89.4°. The sheared perovskite structure is frequently reported as a pseudo-cubic (pc) structure of BFO^5^. In the pseudo-cubic structure, the polarisation axis is parallel to a crystallographic direction <111>, which is thus the polar (i.e. optic) axis. In such a case, four variants of ferroelastic domains and eight degenerated states for the electric polarisation (eight variants of ferroelectric domains taking also the polarisation orientation into account) are expected^8,9^. The knowledge and the control of the domain configurations are essential for the field of “domain engineering” which is striving for tuning the functionalities of devices based on BFO^10,11^. Raman scattering yields information on lattice dynamics. Thus, it is an established method to detect subtle structural changes in BFO^12–15^. For the BFO with the space group ***R3c***, group theory predicts 27 optical phonon modes ${{\rm{\Gamma }}}_{opt,R3c}=4{A}_{1}+5{A}_{2}+9E$. The **A**_**1**_ and doubly degenerated **E** modes are both Raman and IR active, while the **A**_**2**_ modes are silent. This means that 13 modes are observable by Raman spectroscopy from (111)_pc_ BFO scattering surface^16–18^. The Raman tensors of the Raman modes are given by^19^:

1 $${A}_{1}(z)=(\begin{array}{ccc}a &  & \\  & a & \\  &  & b\end{array}),\,E(x)=(\begin{array}{ccc} & c & d\\ c &  & \\ d &  & \end{array}),\,E(y)=(\begin{array}{ccc}c &  & \\  & -c & d\\  & d & \end{array})$$

The modes are polarised along the directions x, y, or z, as shown the inset on the right-hand side of Fig. 1; z denotes the optic axis parallel to the [111]_pc_ direction; y||σ_v_||[$1\overline{2}1$]_pc_. **A**_**1**_ and **E** are polar modes, thus, due to the macroscopic electric field, they split into longitudinal optical (LO) and transversal optical (TO) modes with different frequencies. According to the group theory, polar phonon modes with A_1_ symmetry are polarised along the z-direction (i.e. optic axis //(111)_pc_), while in the case of vibrations with E symmetry, the atomic displacement is perpendicular to the z-axis. The frequency of the polar modes depends on the direction of the phonon propagation with respect to the optic axis, so that a directional dispersion occurs^20–22^. For this reason the oblique modes (propagating along a direction which forms an angle θ with the [111]_pc_ optic axis) have usually a mixed symmetry character^23,24^. Consequently, due to the LO/TO splitting 22 modes should be observed by Raman spectroscopy from scattering surfaces different from (111)_pc_. This is also the case for backscattering from the (001)_pc_ oriented surface of BFO, for which θ = 54.7°. Unfortunately, this aspect was neglected in some of the Raman analyses performed on the (001)_pc_ oriented BFO, which yielded to some controversies in the mode assignments^25–28^. A full assignment of the modes considering mixed symmetry LO/TO and mixed character A_1_/E was done by Hlinka *et al*. based on dispersion of phonons frequency on the angle θ (angle between the phonon propagation and the optic axis)^24^. The method presented in ref.^24^ is based on the phonon frequency variation with the angle between the crystal optic axis and the laser beam in randomly oriented crystalline grains.

In this work we present a method based on the Raman tensor formalism, which allows an assignment of the BFO Raman modes of pure as well as mixed character/symmetry. It should be mentioned that the Raman tensor approach was only applied until now for pure (not mixed) modes. This restriction corresponds to the cases θ = 0° and θ = 90° (where θ is the angle between the phonon propagation and optic axis), *i*.*e*. the case of (111)_pc_ oriented BFO. Most of the samples/films investigated in literature were, however, (001)_pc_ oriented BFO corresponding to θ = 54.7°, which leads to an incorrect interpretation, because in this case mixed character/symmetry of the modes needs to be considered. The method presented in this work analyses the in-plane (*i*.*e*. in the crystal surface plane) variation of the Raman intensities upon azimuthal rotation of a single crystal instead of the phonon frequency dispersion (which was reported in ref.^24^). Since it is based on the Raman intensity analysis, the Raman tensor method presented here is highly sensitive to the in-plane orientation of the optic axis projection onto the sample surface for any angle θ between the phonon propagation (laser beam direction) and the optic axis. Using the 180° periodicity of the azimuthal angular dependence of the intensity of some BFO Raman modes, this method allows for identification of the ferroelastic domains. Thanks to the sensitivity of the Raman signal to the domain orientation that is combined with the depth information when using two appropriate laser wavelengths for excitation, Raman spectroscopy can be used for domain imaging in BFO, as an alternative to other methods such as birefringence^29^ or circular dichroism photoemission^30^.

## Material and Methods

The samples investigated in this work were 150 µm thick (001)_pc_ oriented BFO crystals containing macroscopic ferroelastic domains. The crystals were grown in Bi_2_O_3_ + B_2_O_3_ flux using a method similar to that originally proposed by Kubel and Schmid^31^. The top solution crystals were harvested and polished parallel to the (001)_pc_ surface. The final polishing was performed using a SiO_2_ colloidal solution diluted with water in a 1:1 ratio in order to remove residual mechanical strain.

### Raman spectroscopy

Raman spectroscopic measurements were performed in backscattering geometry at room temperature using Horiba Jobin Yvon HR 800 spectrometers equipped with 2400 mm^−1^ or 1800 mm^−1^ gratings. The 442 nm line of a HeCd laser and the 633 nm line of a HeNe laser were used for excitation. The light was focused and collected through a 50x magnification objective (about 2 µm focus diameter). The laser power was kept below 1.5 mW in order to avoid changes in the Raman spectra induced by laser heating. The light of the incident and scattered beam was polarised either parallel (VV) or perpendicular (HV) to each other.

### Electron backscatter diffraction

The distribution of ferroelastic domains was verified by electron backscatter diffraction (EBSD) measurements that were performed on a scanning electron microscope (Zeiss LEO 1530 GEMINI) operating at an acceleration voltage of 20 kV. The Kikuchi patterns were recorded with a Nordlys II detector (Oxford Instruments) at the step size of 0.2 µm, and subsequently indexed by the HKL Channel 5 software. For indexing, the crystal structure suggested by Moreau *et al*.^6^ was used. The EBSD measurements confirmed the almost single-crystalline nature of the BFO crystals and their orientation (001)_pc_ parallel with the sample surface. Because of the pseudo-cubic symmetry of BFO and the centrosymmetry of the EBSD patterns, EBSD cannot distinguish easily between the orientation variants, which are mutually rotated about approximately 90°, 180° or 270° around the axis perpendicular to the sample surface^32^. Still in this particular case, the EBSD measurement was able to trace the local orientation of the polarisation axis through the elastic distortion of the crystal structure that is caused by the polarisation/ferroelastic effect. Thus, the smallest mean angular deviation (MAD) between the measured and simulated Kikuchi patterns of BFO was utilized as a decision criterion for the respective orientation variant and for the corresponding orientation of the polarisation axis.

## Results and Discussions

### Raman spectroscopy. Assignment of the oblique phonon modes

The inset in the middle of Fig. 1 shows a polarisation optical microscopy image of one of the BFO crystals (ca. 250 µm × 500 µm) investigated in this work, presenting two large homogeneous ferroelastic domains: a dark diagonal stripe and the remainder of the sample (further denoted as large domain). The optical contrast between the two domains is due to different orientations of the spontaneous electric polarisation. In both cases the spontaneous polarisation lies along a <111>_pc_ diagonal, but the two polarisation projections on the sample surface are forming an angle of 90° with one another. The contrast of the image can be reverted by rotating the sample by 90° around the surface normal under the polarisation microscope and keeping the polariser and the analyser of the polarisation microscope fixed. Piezoresponse force microscopy (see Supplementary Material, Fig. 1s) indicates that for both domains the polarisation points downwards along <111 > _pc_ diagonal, which means that 71° domain walls should be formed^31,33,34^.

Figure 1 shows characteristic Raman spectra measured on the two different domain regions, which are marked by asterisks in the inset, with the analyser being parallel to the polarisation of the incident light (VV). The spectra were excited with a laser line of 633 nm, which ensures non-resonant conditions and a relatively high integrated intensity of the first order Raman signal at room temperature^35^. On one hand the 633 nm excitation allows the use of the Raman tensor formalism for the assignment of Raman modes. On the other hand, the penetration depth of the laser light is very large because of the low extinction coefficient *k* = 1.2 × 10^−4^ ^36,37^. Thus, BFO is nearly transparent for the 633 nm light; the excited volume is actually given by the focus depth (~7 µm). The Raman spectra measured on the stripe domain and on the remainder of the sample (large domain) have very different relative intensities. However, considering a flat (001)_pc_ scattering surface of the sample (as in our case), one expects for both types of domains the same angle θ = 54.7° formed by the direction of laser light propagation and the optic axis. This is confirmed by the fact that the peaks have the same frequency position, as no angular dispersion is expected in this case^22,24^. Therefore the differences between the spectra are given by the different in-plane orientation of the optic axis in the two types of domains. In order to check this in detail Raman measurements have been performed by rotating the sample azimuthally around the laser beam direction (keeping the measurement point) every 15° for both parallel (VV) and crossed (HV) polarisation configurations, and on both types of domains.

**Figure 2:**
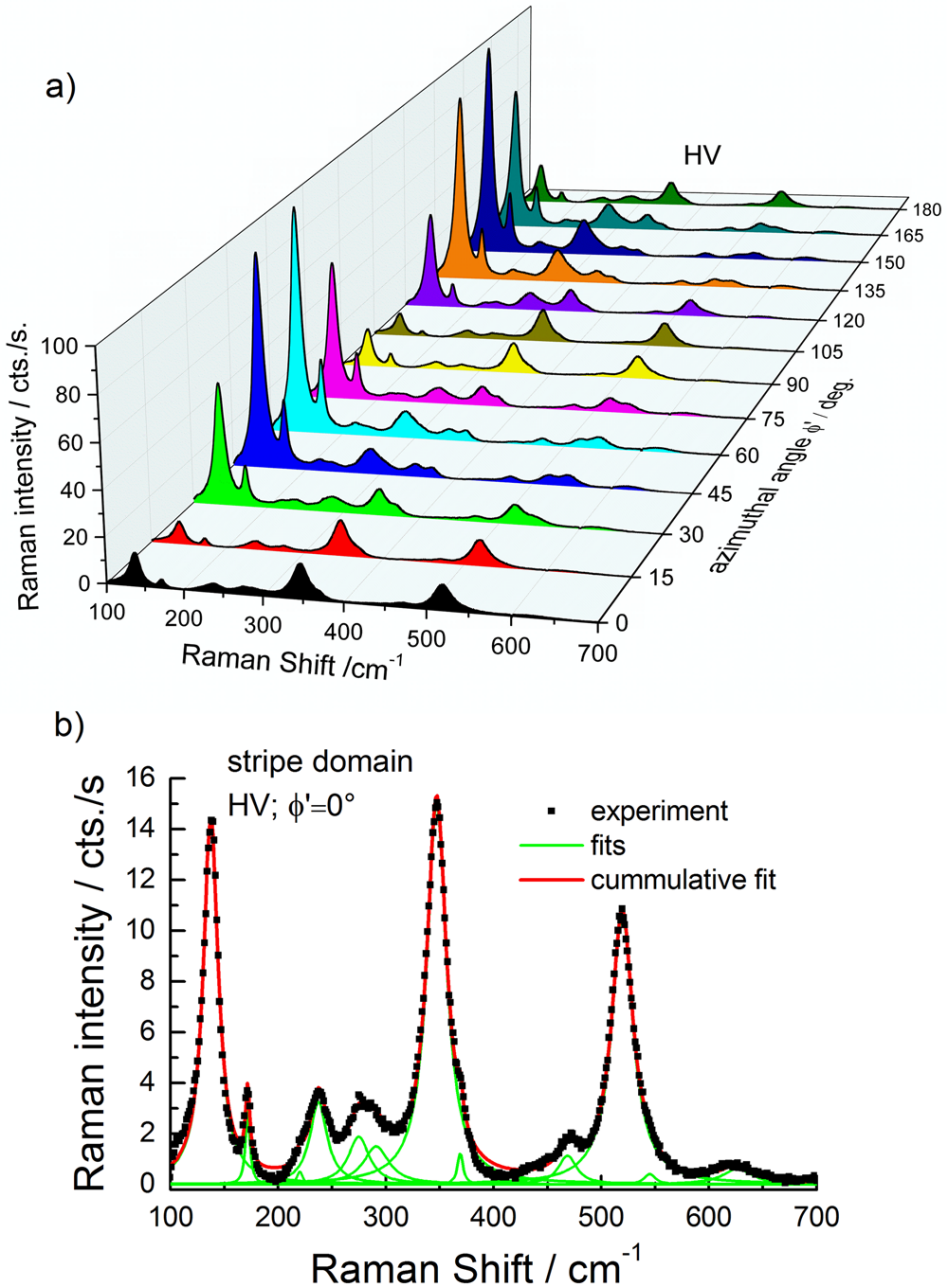
(**a**) Raman spectra measured in crossed (HV) polarisation on the stripe domain for different azimuthal rotation angles ϕ′. (**b**) Example of spectrum evaluation (fit) for ϕ′ = 0°.

**Figure 3:**
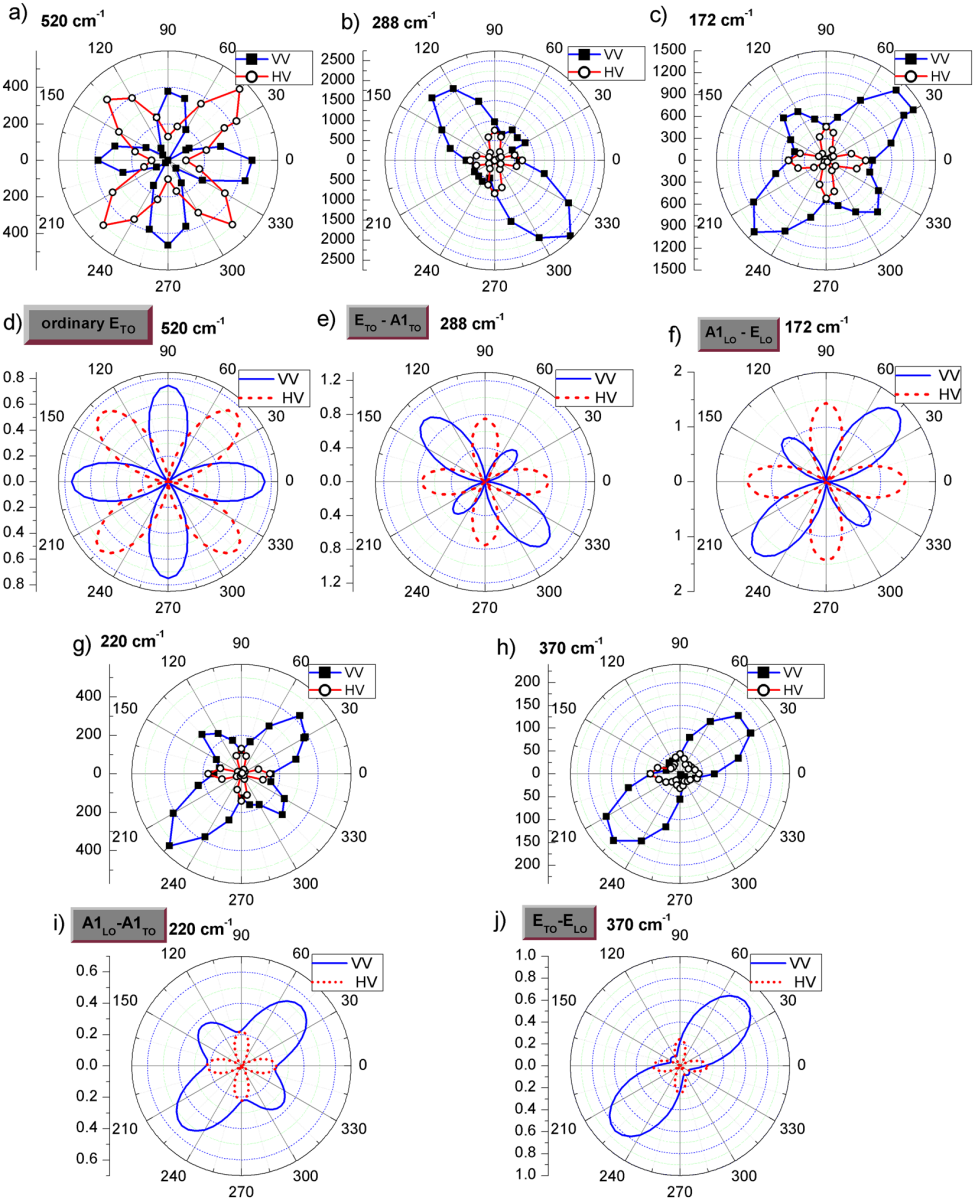
Polar plots of the azimuthal angle (ϕ) dependence of the peak areas for the modes at 520 cm^−1^ (**a**), 288 cm^−1^ (**b**), 172 cm^−1^ (**c**) 220 cm^−1^ (**g**) and 370 cm^−1^ (**h**) measured on the stripe domain using parallel (squares) and crossed (circles) polarisation configurations. Simulated intensity dependence using Raman tensor formalism for the ordinary E_TO_ (**d**), E_TO_↔A1_TO_ (**e**), A1_LO_↔E_LO_ (**f**), A1_LO_↔A1_TO_ (**i**) and E_TO_↔E_LO_ (**j**) mixed modes in the case of an (001)_pc_ BFO scattering surface, for parallel (continuous line) and crossed (dashed line) polarisation configurations.

Figure 2a) shows the spectra measured in HV polarisation configuration on the azimuthally rotated stripe domain. It can be seen that most of the Raman modes show a 90° periodicity of the intensity as a function of azimuthal rotation angle ϕ′. The azimuthal rotation angle ϕ = ϕ′ + 30° is the angle between the [010]_pc_ BFO crystal direction and the direction of the polarisation of the incident laser light. For ϕ = 0° the [010]_pc_ BFO crystal direction is parallel to the polarisation of the light. In order to obtain the intensity angular dependence of the modes each spectrum has been fitted using Lorentzian functions. In Fig. 2b) the fit obtained for the first spectrum in Fig. 2a), measured for ϕ′ = 0° and HV polarisation, is shown as an example. The same procedure was applied for all rotation angles, both HV and VV polarisation configurations, and both domains. In all cases the peak areas depend periodically on the azimuthal angle. Exemplarily, the polar dependence of the peak areas for the peaks located at 520 cm^−1^, 288 cm^−1^, 172 cm^−1^, 220 cm^−1^ and 370 cm^−1^ is plotted in Fig. 3a–c,g and h) respectively, for both VV (squares) und HV (circles) polarisation configurations and for the measurements performed on the stripe domain. We chose to present these modes because they have a high enough intensity for both polarisation configurations. Moreover, their positions are well separated from that of neighbouring modes. The peak area of the modes at 172 cm^−1^, 288 cm^−1^, and 370 cm^−1^ shows a 180° periodicity for VV polarisation and a 90° periodicity for HV polarisation. In case of the 520 cm^−1^ mode a periodicity of 90° was obtained for both polarisation configurations. The polar dependence of the intensity of the peaks when measuring on the large domain is rotated by 90° as compared with the measurements on the stripe domain. A comparison of the intensity for the modes at 138 cm^−1^ and at 370 cm^−1^ on stripe and large domains is exemplarily shown in the Fig. 2s in the Supplementary material. The anisotropic behaviour (180° periodicity) of the intensity allows us to distinguish between the macroscopic ferroelastic domains as will be shown in the line scans and maps discussed in the next section of the paper.

As mentioned above, for a (001)_pc_ scattering surface the laser beam direction has an angle of θ = 54.7° with respect to the direction of the spontaneous polarisation, which is along a < 111 > _pc_ direction (parallel to the optic axis). In this case, as discussed in the introduction, the polar **E** and **A**_**1**_ modes show a directional dispersion as function of θ^24^. Besides the ordinary **E**_TO_ modes, whose frequencies are independent on the angle θ, there are four types of extraordinary modes with mixed symmetries: **E**_TO_↔**E**_LO_, **E**_TO_↔**A**_**1**TO_, **A**_**1**LO_↔**A**_**1**TO_, and **A**_**1**LO_↔**E**_LO_^38^. For the assignment of these oblique phonon modes the mixed character of the modes which correspond to θ = 54.7° should be taken into account.

The Raman tensor formalism allows for consideration of mixed phonon mode character based on a unit vector of phonon polarisation (cf. Yang *et al*.^39^ and Talkenberger *et al*.^38^). For calculation of the Raman scattering intensity as function of the azimuthal angle, equation (9) from Talkenberger *et al*.^38^ and the corresponding Raman tensors (equation 1) were used. The unit vectors of incident and scattered light as well as the phonon polarisation of ordinary and extraordinary modes were expressed with respect to the crystal (x,y,z) coordinate system assuming backscattering geometry. Using the method described above the azimuthal dependence of the intensity of all symmetries: ordinary E_TO_, E_TO_↔A_1__TO_, A_1__LO_↔E_LO_, A_1__LO_↔A_1__TO_ and E_TO_↔E_LO_ were simulated for both VV and HV polarisation configurations in agreement with the experimental data as shown in Fig. 3d–f,i,j), respectively. It should be mentioned that each specific vibration has unique tensor elements. For their accurate determination, crystals with several differently oriented scattering surfaces would be needed. However, using the values of the Raman tensor elements given in the Table 1, a very good agreement between the simulated and the experimental data could be obtained for all five symmetries as shown in Fig. 3. At a closer look, when comparing experimental and simulated data, one can see that some of the experimental curves in parallel polarisation are not going to zero intensity as some of the simulated curves do. Possible reasons for this may be: the used angular step size of 15° could skip the very narrow minimum in intensity, the correlation errors in the fitting procedures (especially for neighbouring modes), a slight tilt of the sample surface, and/or polarisation leakages which are softening the selection rules. Nevertheless, our method allows a clear assignment of mixed symmetries of most of the BFO modes in the case of (001)_pc_ scattering surfaces. The azimuthal dependence of the 220 cm^−1^ and the 172 cm^−1^ modes looks similar. In this case the phonon dispersion (where only one mode at 220 cm^−1^ has A_1__LO_-A_1__TO_ mixed symmetry)^24,38^, has to be used to distinguish between their symmetry. It should be mentioned that for all the modes mentioned in Table 1 the same symmetry assignment was found as expected from the dispersion curves from Hlinka *et al*.^24^ for θ = 54.7° which corresponds to our (001)_pc_ oriented BFO.

**Table 1:** The modes and their assigned symmetry.

Symmetry	Modes	Tensor elements
Ordinary **E**_**TO**_	520 cm^−1^, 237 cm^−1^, 347 cm^−1^	c_TO_ = 0.2, d_TO_ = 1.2
**E**_**TO**_ **-A**_**1**__**TO**_	288 cm^−1^, 138 cm^−1^, 545 cm^−1^	a_TO_ = −1.1, b_TO_ = 0.6 c_TO_ = 0.2, d_TO_ = 1.2
**A**_**1**__**LO**_ **-E**_**LO**_	172 cm^−1^, 470 cm^−1^, 620 cm^−1^	a_LO_ = 0.4, b_LO_ = 0.4 c_LO_ = 1.0, d_LO_ = 1.7
**A**_**1**__**LO**_ **-A**_**1**__**TO**_	220 cm^−1^	a_LO_ = −1.0, b_LO_ = 1.0, a_TO_ = 0.2, b_TO_ = 1.2
**E**_**TO**_ **-E**_**LO**_	370 cm^−1^, 432 cm^−1^	c_LO_ = 0.2, d_LO_ = 1.0, c_TO_ = −0.5, d_TO_ = 0.9

The tensor elements correspond to the phonon modes for which the simulations were shown in Fig. 3 (the first mode in each table line).

### Imaging of the ferroelastic domains

**Figure 4:**
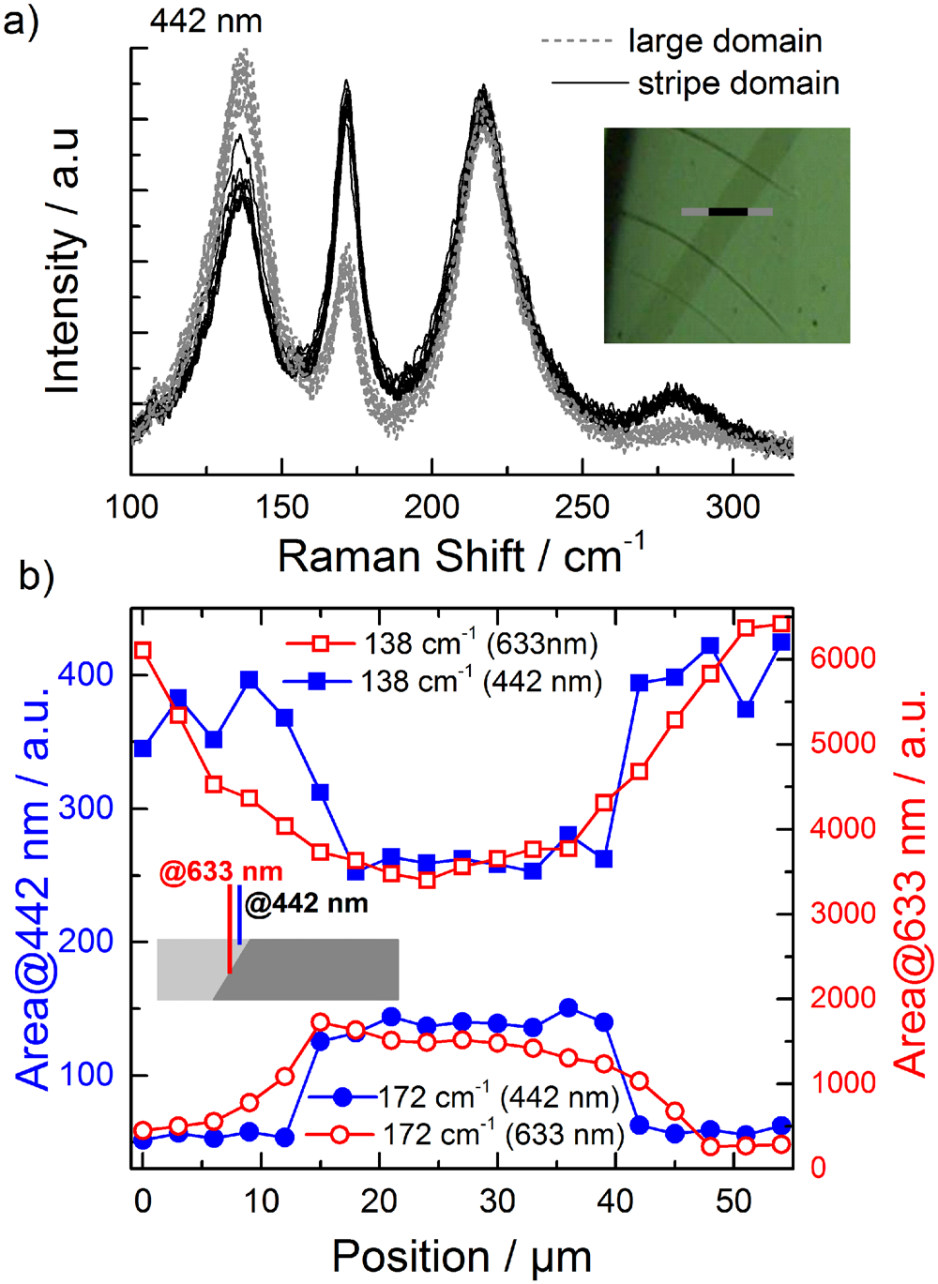
(**a**) Raman spectra measured using 442 nm in a line scan crossing the stripe domain. The spectra measured on the large domain are plotted by dashed lines, and those measured on the stripe domain by continuous lines. (**b**) The peak areas of the 138 cm^−1^ and 172 cm^−1^ modes show well separated stripe and large domains when measuring with 442 nm excitation line (full symbols). The interface region shows a continuous change in the peak areas when measuring with 633 nm excitation line (open symbols). The inset shows schematically the interface between the two domain types.

Using the 180° periodicity of the azimuthal angle dependence of the modes at 138 cm^−1^ (**E**_TO_↔**A**_**1**TO_) and 172 cm^−1^ (**A**_**1**LO_↔**E**_LO_) one can now use Raman spectroscopy for a quick distinction between different domains. A Raman spectroscopic line scan was realised along a 54 µm line crossing the stripe domain, with measurements done every 3 µm as can be seen in the inset of Fig. 4a). In the same figure, the Raman spectra measured with the 442 nm excitation line in parallel polarisation (VV) are shown. The grey dotted spectra correspond to points measured on the large domain, while the black continuous spectra are measured on the stripe domain. The 442 nm (2.81 eV) excitation line is slightly above the BFO bandgap of ~2.75 eV which means that near-resonant Raman scattering can be observed when using this line. In such conditions a breakdown of selection rules can occur which can affect the intensity of the modes^40–42^. In order to exclude any ambiguity in the interpretation of the mode characters, the Raman tensor formalism combined with azimuthal dependent measurements was only applied for the case of off-resonant measurement performed with 633 nm (1.96 eV). Once the mode characters have been unambiguously determined, one can take advantage of the different penetration depth of the two laser lines in BFO (76 nm @442 nm and 7 µm @633 nm)^37^. Thus, line scan measurements were performed using both excitations in order to obtain depth information on the domain walls. Considering the low penetration depth of the light for 442 nm excitation these measurements are very sensitive to the surface of the sample. The spectra have been evaluated using a similar fitting procedure as for the azimuthal angle dependent measurements. In Fig. 4b) the peak areas for the modes at 138 cm^−1^ and 172 cm^−1^ are plotted by full symbols. It can be seen that the intensities (as peak areas) of these two peaks can be used to identify the two types of ferroelastic domains showing a sharp interface (in the limit of the 3 µm steps) between them.

The same measurement procedure was carried out in addition using the 633 nm laser as an excitation source. As discussed previously, the extinction coefficient is very low for this wavelength, thus the information depth is given by the focus depth (~7 µm). Under such conditions, the information obtained from the experiment is not limited to the surface of the sample, but stems also from the “bulk”. For this reason, the transition at the domain wall between the intensities (as peak areas) of the two Raman modes measured with 633 nm (open symbols in Fig. 4b)) is not as sharp as for the 442 nm excitation. This indicates that the interfaces between the two neighbouring domains are not vertical but inclined, as schematically shown in the inset of the figure. Such inclined domain walls are typically formed in the case of so called 71° domain pattern^10,34,43^, as was confirmed by piezoresponse force microscopy (Fig. 1s, Supplementary Material). Thus, the Raman spectroscopy offers not only a tool for distinguishing between differently oriented ferroelastic domains in BFO crystals, but is also capable to supply information about the depth profile and shape of the domain interfaces if different excitation wavelengths are used.

**Figure 5:**
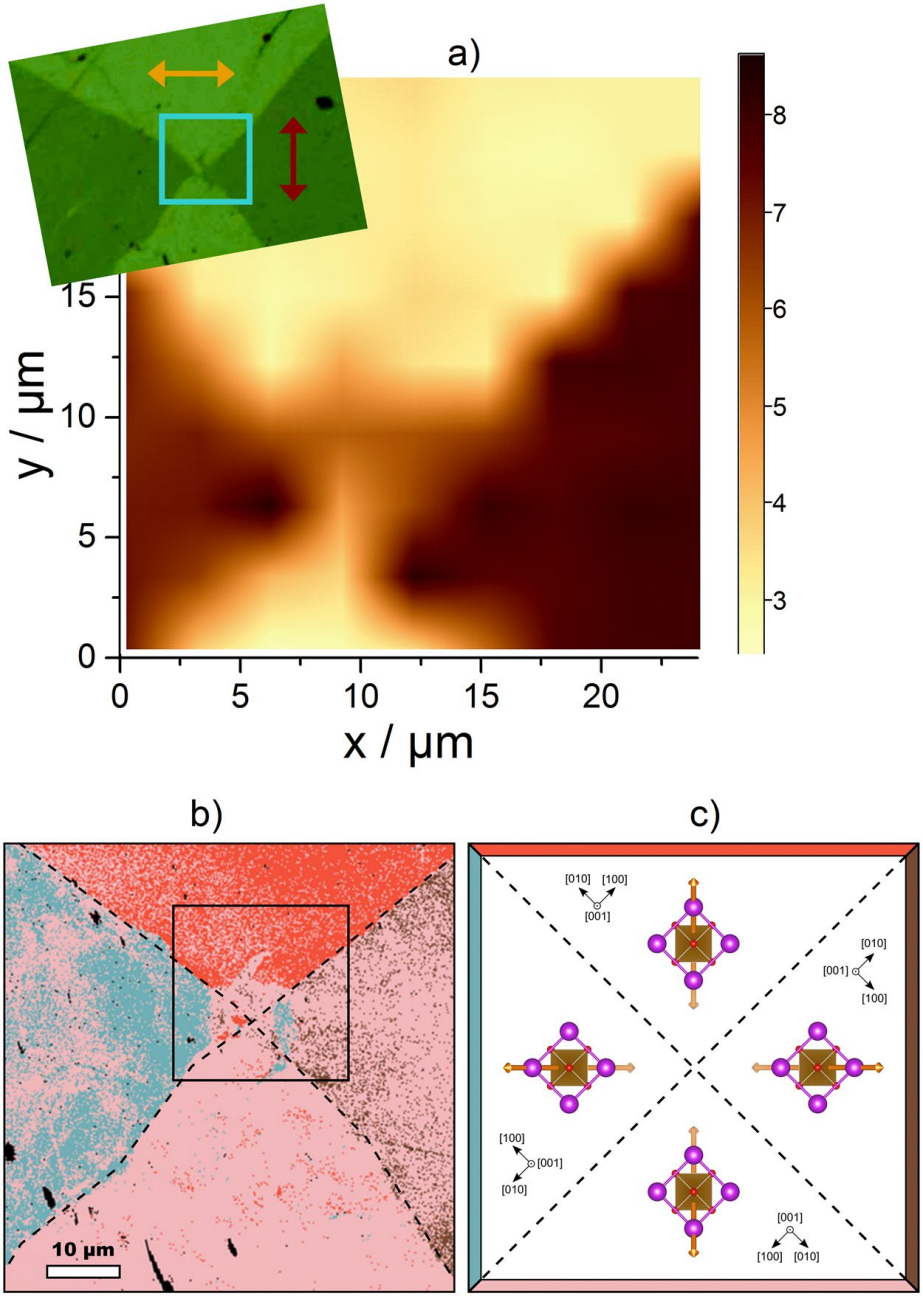
(**a**) Intensity of the 172 cm^−1^ mode shown as Raman map on an area of 24 µm × 24 µm (3 µm steps) indicated by the square in the polarisation microscopy image (in the top-left side). The arrows indicate the projections of the spontaneous polarisation on the sample surface. (**b**) Local EBSD map and (**c**) the corresponding idealized schematic domain structure. The domain boundaries are highlighted by dashed lines. The area marked in (**b**) by black square corresponds to the Raman map presented in (**a**). All four domains contain regions with crystallographically equivalent orientations. Different colours of the spots (red, brown, pink and blue) result from different local rhombohedral distortions that are associated with different orientations of the polarisation axis. The polarisation axes are depicted by orange arrows in figure (**c**), where also the dominant orientation variants are illustrated.

The Raman line scans can easily be extended to two-dimensional Raman mapping. In the upper left part of the Fig. 5a) macroscopic ferroelastic domains forming a diagonally tiled structure are revealed by polarised light microscopy on another BFO sample. The arrows in the polarisation microscopy image correspond to the projection of the spontaneous polarisation for the two domain types on the sample surface and they are perpendicular to each other. Raman mapping on this sample was carried out on an area of 24 µm × 24 µm indicated by the square, with a step size of 3 µm between measurements using the 442 nm laser for excitation. Each single Raman spectrum was fitted as described previously. The integral intensity (peak area) of the 172 cm^−1^ peak, that shows 180° periodicity of the azimuthal angle dependence in VV polarisation in Fig. 3c), is plotted in the Fig. 5a) as a map. In the intensity map a very clear distinction between the two types of domains can be seen. The shape of the Raman map is very similar compared to that of the polarisation microscopy image, which shows again the capability of Raman spectroscopy to distinguish between different ferroelastic domains.

The results of the optical polarisation microscopy and the Raman map (Fig. 5a)) were supported by local EBSD measurements (Fig. 5b)). As the smallest MAD value was employed as the main criterion for the selection of the respective orientation variant by means of EBSD, the different colours stand for different local rhombohedral distortions of the pseudo-cubic cell of BFO that are associated with different macroscopic orientations of the polarisation axis [111]_pc_. Generally, four different orientations of BFO were detected in the examined area (red, brown, pink and blue, see Fig. 5b)), which were assigned to domains with idealized orientations as shown in Fig. 5c). These domains are rotated by 0°, 90°, 180° and 270° with respect to a laboratory coordinate system. However, as the rhombohedral distortion is small and close to the resolution limit of EBSD, the domain assignment using MAD is complicated by various microstructure phenomena and defects. This leads to an apparent switching between individual orientations of the polarisation axis within a domain. Nevertheless, the domain structure pattern revealed by the EBSD measurement agrees well with the domain structure arrangement obtained from the polarisation microscopy image and from the Raman mapping, where the projected polarisation axes of the neighbouring and opposing domains were approximately perpendicular and parallel, respectively, cf. Fig. 5a). This finding is consistent with the domain structure, which is typically reported in the BFO domains having the surface orientation {001}_pc_^33^. A good agreement between the EBSD map and polarisation microscopy image was achieved even in the interface regions with a complex domain structure. A small tail penetrating into the upper domain that was detected by the polarisation microscopy (Fig. 5a)) was also very well reproduced by the EBSD mapping (Fig. 5b)).

## Conclusions

In summary, we present a general approach based on numerical simulations using the Raman tensor formalism combined with polarisation and azimuthal angle-dependent Raman measurements that is capable to identify the polar modes of mixed symmetries in a (001)_pc_ oriented BFO single crystal unambiguously. This method can easily be extended to any crystal orientation. The intensities of some modes are sensitive to the arrangement (in-plane orientation) of the ferroelastic domains. The anisotropic 180° periodicity of the azimuthal angular dependence of their intensity in parallel polarisation can be used for quick identification of the domains and for mapping large domains in BFO, even though the spatial resolution and the scan velocity is low. An advantage of Raman mapping/line scans performed at different excitation wavelengths having different light penetration depths in BFO is the depth sensitivity of the information regarding the tilt of the domain walls relative to the surface.

## Electronic supplementary material

Supplementary Material
