# Supplementary material for: Ferroelastic domain identification in BiFeO3 crystals using Raman spectroscopy
Source: Sci Rep. 2019 Jan 23;9:379. doi: 10.1038/s41598-018-36462-5 (PMC6344528; doi:10.1038/s41598-018-36462-5)
Supplement: Supplementary file 1 — Supplementary Material [file 41598_2018_36462_MOESM1_ESM.doc]

Supplementary online material

**Ferroelastic domain identification in BiFeO_3_ crystals using Raman spectroscopy**

Cameliu Himcinschi^1*^, Jan Rix^1^, Christian Röder^1^, Martin Rudolph^2^, Ming-Min Yang^3^, David Rafaja^2^, Jens Kortus^1^, and Marin Alexe^3^

* himcinsc@physik.tu-freiberg.de

Supplementary Figure 1s


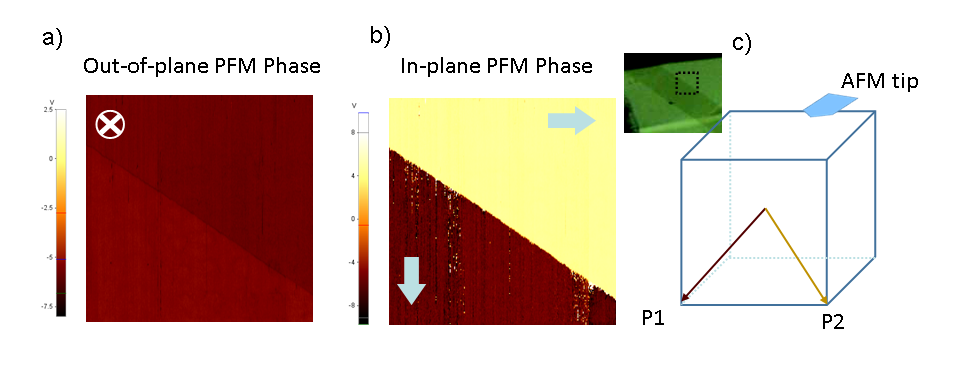


(a) Out-of-plane piezoresponse force microscopy (PFM) phase signal and (b) in-plane PFM phase signal of a 15 µm x15 µm region marked by the square in the inset of optical microscopy image of figure (c). The measured region contains both types of ferroelastic domains: dark diagonal stripe and the remainder of the sample. PFM measurements indicate that for both types of domains the ferroelectric polarisation points downwards, so that 71° -type ferroelectric domains are formed.

Supplementary Figure 2s

| 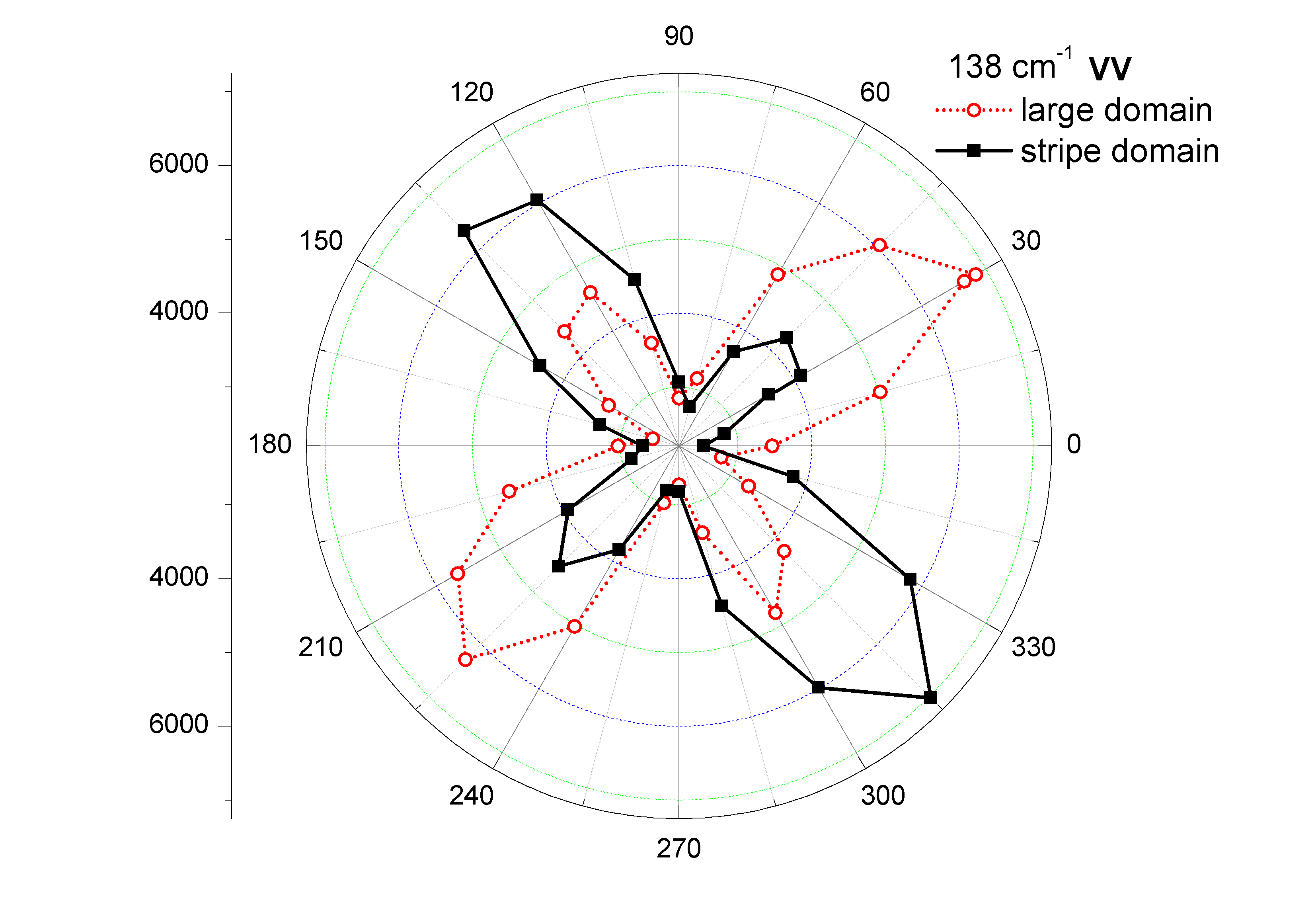 | 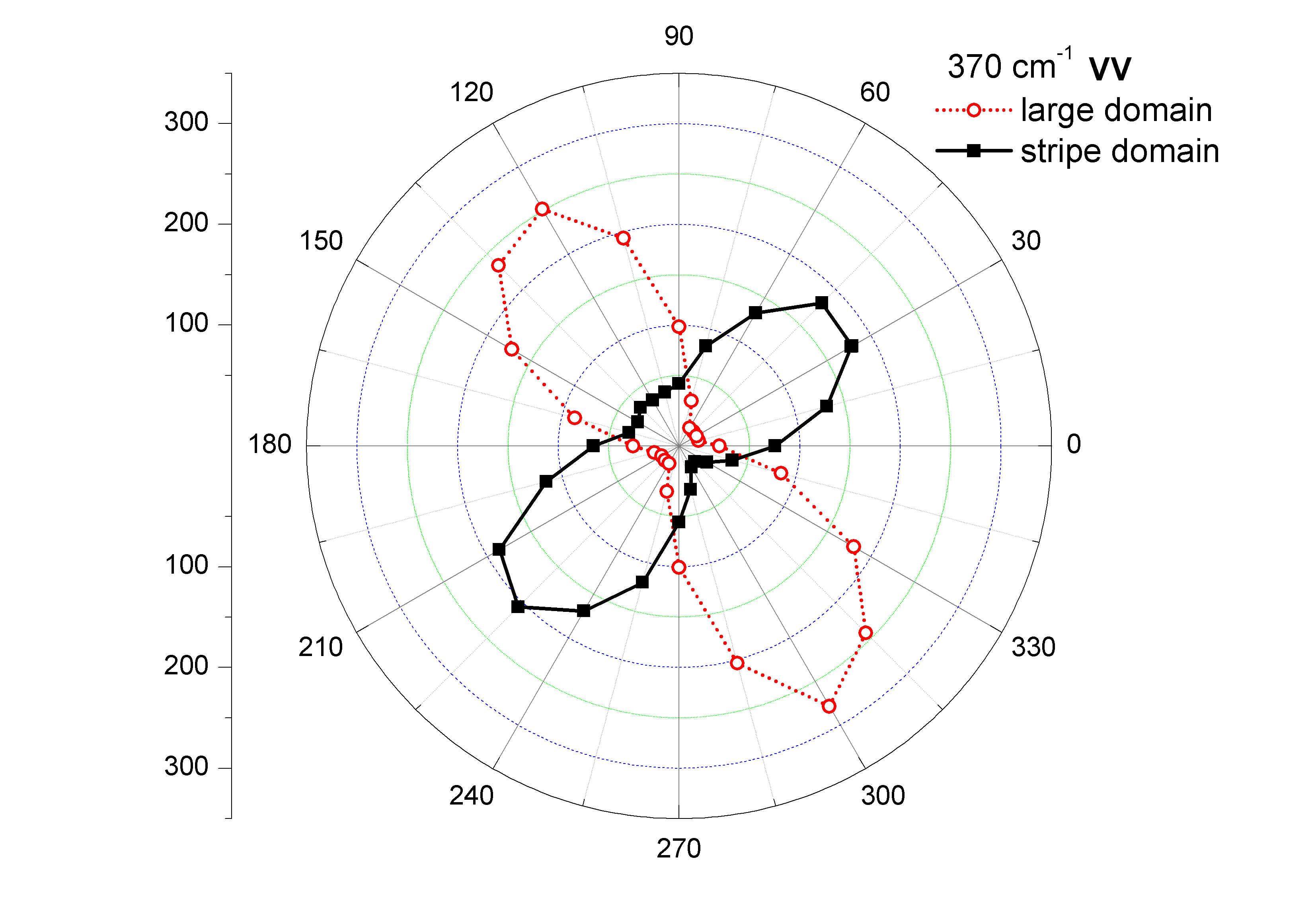 |
| --- | --- |
| Polar plots of the azimuthal angular dependence of the peak area for the modes at 138 cm^-1^ (left side) and at 370 cm^-1^ (right side) measured using parallel (VV) polarisation configuration on the large domain (circles) and on the stripe domain (squares). The anisotropic behaviour (180° periodicity) of the intensity was used to distinguish between the macroscopic ferroelastic domains as discussed in the paper. | |
